# A Novel Strategy for the Detection of SARS-CoV-2 Variants Based on Multiplex PCR-Mass Spectrometry Minisequencing Technology

**DOI:** 10.1128/Spectrum.01267-21

**Published:** 2021-11-17

**Authors:** Fei Zhao, Jinxing Lu, Bing Lu, Tian Qin, Xuemei Wang, Xuexin Hou, Fanliang Meng, Xiaona Xu, Tianyi Li, Haijian Zhou, Jianzhong Zhang, Biao Kan, Ying Huang, Zehui Zhang, Di Xiao

**Affiliations:** a National Institute for Communicable Disease Control and Prevention, Chinese Center for Disease Control and Prevention, State Key Laboratory of Infectious Disease Prevention and Control, Beijing, China; b Institute for Infectious Disease and Endemic Disease Control, Beijing Center for Disease Prevention and Control, Beijing Research Center for Preventive Medicine, Beijing Key Laboratory of Diagnostic and Traceability Technologies for Food Poisoning, Beijing, China; c Intelligene Biosystems (Qingdao) Co., Ltd., Qingdao, China; Peking University People’s Hospital

**Keywords:** SARS-CoV-2 variants, SNPs, screening and identification, mPCR-MS minisequencing

## Abstract

The objective of this study was to construct a novel strategy for the detection of severe acute respiratory syndrome coronavirus 2 (SARS-CoV-2) variants using multiplex PCR-mass spectrometry minisequencing technique (mPCR-MS minisequencing). Using the nucleic acid sequence of a SARS-CoV-2 nonvariant and a synthetic SARS-CoV-2 variant-carrying plasmid, a matrix-assisted laser desorption ionization–time of flight mass spectrometry (MALDI-TOF MS) method based on the single-base mass probe extension of multiplex PCR amplification products was established to detect 9 mutation types in 7 mutated sites (HV6970del, N501Y, K417N, P681H, D614G, E484K, L452R, E484Q, and P681R) in the receptor-binding domain of the spike protein of SARS-CoV-2 variants. Twenty-one respiratory tract pathogens (9 bacteria and 12 respiratory viruses) and nucleic acid samples from non-COVID-19 patients were selected for specific validation. Twenty samples from COVID-19 patients were used to verify the accuracy of this method. The 9 mutation types could be detected simultaneously by triple PCR amplification coupled with MALDI-TOF MS. SARS-CoV-2 and six variants, B.1.1.7 (Alpha), B.1.351 (Beta), B.1.429 (Epsilon), B.1.526 (Iota), P.1 (Gamma) and B.1.617.2 (Delta), could be identified. The detection limit for all 9 sites was 1.5 × 10^3^ copies. The specificity of this method was 100%, and the accuracy of real-time PCR cycle threshold (*C_T_*) values less than 27 among positive samples was 100%. This method is open and extensible, and can be used in a high-throughput manner, easily allowing the addition of new mutation sites as needed to identify and track new SARS-CoV-2 variants as they emerge. mPCR-MS minisequencing provides a new detection option with practical application value for SARS-CoV-2 and its variant infection.

**IMPORTANCE** The emergence of SARS-CoV-2 variants is the key factor in the second wave of the COVID-19 pandemic. An all-in-one SARS-CoV-2 variant identification method based on a multiplex PCR-mass spectrometry minisequencing system was developed in this study. Six SARS-CoV-2 variants (Alpha, Beta, Epsilon, Iota, Gamma, and Delta) can be identified simultaneously. This method can not only achieve the multisite simultaneous detection that cannot be realized by PCR coupled with first-generation sequencing technology and quantitative PCR (qPCR) technology but also avoid the shortcomings of time-consuming, high-cost, and high technical requirements of whole-genome sequencing technology. As a simple screening assay for monitoring the emergence and spread of SARS-CoV-2 and variants, mPCR-MS minisequencing is expected to play an important role in the detection and monitoring of SARS-CoV-2 infection as a supplementary technology.

## INTRODUCTION

Severe acute respiratory syndrome coronavirus 2 (SARS-CoV-2) has spread worldwide since the end of 2019. The second wave of COVID-19 has been incessantly causing catastrophe worldwide. The emergence of SARS-CoV-2 variants is the key factor in the second wave of the COVID-19 pandemic. As of June 2021, there are six different SARS-CoV-2 variants have been identified, including the United Kingdom variant (B.1.1.7, Alpha), South African mutant (B.1.351, Beta), California mutant (B.1.429, Epsilon), New York mutant (B.1.526, Iota), Brazilian mutant (P.1, Gamma), and Indian mutant (B.1.617.2, Delta) (1, 2). Some reports suggest that these variants have important mutations in the receptor-binding domain (RBD) of the spike protein of SARS-CoV-2 that increase the transmission efficiency of the virus, increase the severity of the disease, enhance the immune escape ability of the virus, and reduce the immune effect of the current vaccines (3–6); this has not been proven to be universally true.

Currently, the mutation and evolution of SARS-CoV-2 are mainly detected by whole-genome sequencing (WGS) (7–11). Although genome sequencing is the gold standard for identifying SARS-CoV-2 variants, routine genomic testing is expensive and difficult to perform in real time and is not available in many areas due to lack of resources and expertise. Quantitative PCR (qPCR) (12), melting-temperature RT-PCR (13), and CRISPR-Cas13a-based transcription amplification were general used for the single-site detection of SARS-CoV-2 variants (14). Therefore, the rapid, accurate, economic, and multisite detection method of SARS-CoV-2 variant identification is an urgent technical system for SARS-CoV-2 infection prevention and control worldwide.

Matrix-assisted laser desorption ionization–time of flight mass spectrometry (MALDI-TOF MS) has been utilized for detecting single nucleotide polymorphisms (SNPs) in recent decades, and the basic process is as follows. Multiplex PCR is used to amplify the genes containing the targets of SNPs. Subsequently, an extension mass probe is utilized for the extension of SNP sites. Finally, MALDI-TOF MS is performed to identify the mass-to-charge ratio (*m/z*) of extended mass probes. In order to understand and distinguish from other mass spectrometry techniques, we named this technique of detecting SNPs by multiplex PCR coupled with MALDI-TOF MS as multiplex PCR-mass spectrometry minisequencing (mPCR-MS minisequencing) technology. In recent years, MALDI-TOF MS has been commonly used in clinical hospitals, the Center for Disease Control and Prevention, and research institutes in many countries for microbiological detection and analysis (15–17). To date, multiple-SNP detection and diagnosis of various viral infections based on MALDI-TOF MS have been frequently conducted (18–20). Our team has also completed a multisite SNP genotyping and macrolide susceptibility gene method for Mycoplasma pneumoniae using mPCR-MS minisequencing. SNP analysis based on MALDI-TOF MS features high-throughput, rapid detection and simultaneous detection of multiple targets (21). Therefore, it is one of the most promising biomolecular techniques. In this study, 9 mutation types of 7 mutation sites in the RBD of the spike protein were detected simultaneously by using mPCR-MS minisequencing technology for the identification all current SARS-CoV-2 variants.

## RESULTS

### MPCR-MS minisequencing-based method establishment and optimization.

**FIG 1:**
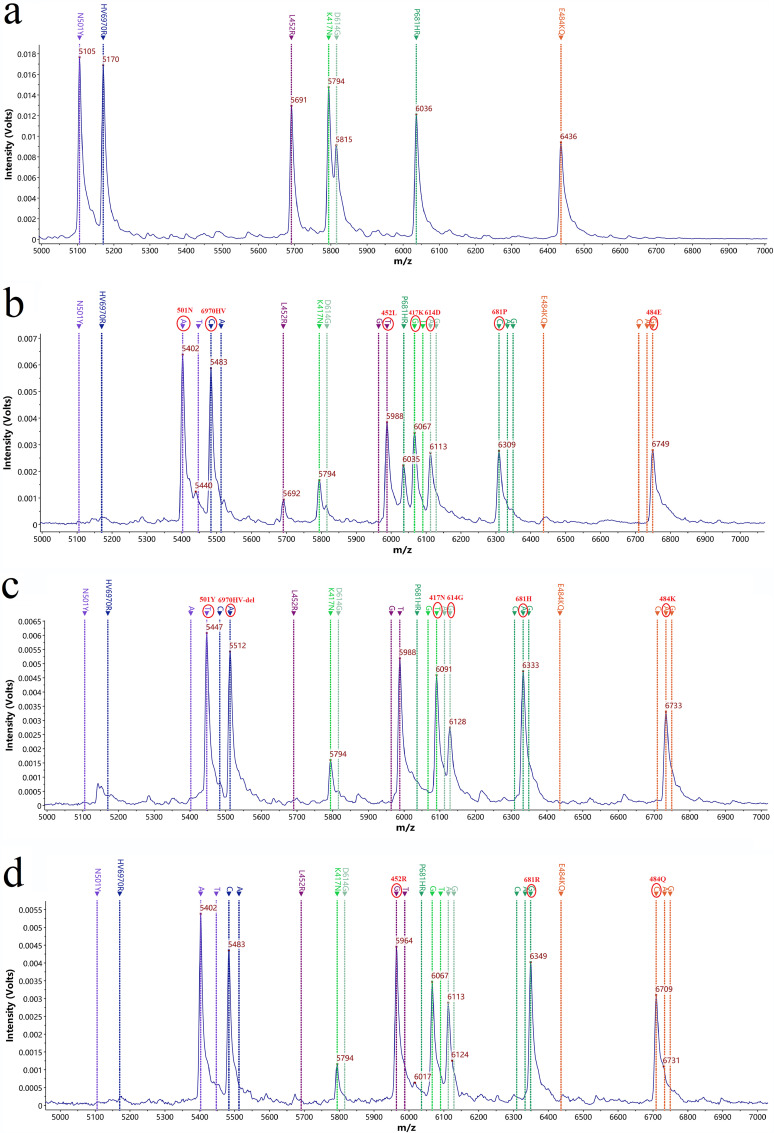
MS peak of the MPE probes. (a) MS peaks of 9 MPE probes without extension; (b) target site peaks of the MPE probes extended to non-SARS-CoV-2 variants; (c) target site peaks of the MPE probes extended to SARS-CoV-2 S gene mutation plasmid 1 (containing the HV69-70del, K417N, E484K, N501Y, D614G, and P681H mutations); (d) target site peaks of the MPE probes extended to SARS-CoV-2 S gene mutation plasmid 2 (containing the L452R, E484Q, and P681R mutations).

The nine targets were amplified by triple PCR. The *m/z* of mass probe extension (MPE) original peaks at 9 mutation types (HV69-70del, N501Y, K417N, P681H, D614G, E484K, L452R, E484Q, and P681R) were 5,170 ± 3, 5,105 ± 3, 5,794 ± 3, 6,036 ± 3, 5,816 ± 3, 6,436 ± 3, 5,691 ± 3, 6,436 ± 3, and 6,036 ± 3, respectively (mass error less than 500 ppm; the same below). The *m/z* of MPE peaks of these 9 mutation types of SARS-CoV-2 nonvariants were 5,483 ± 3, 5,402 ± 3, 6,067 ± 3, 6,309 ± 3, 6,113 ± 3, 6,749 ± 3, 5,988 ± 3, 6,749 ± 3, and 6,309 ± 3, and the extension bases were C, A, G, C, A, G, G, G, and C. The *m/z* of MPE peaks of these 9 mutation types of the S gene-mutated plasmid of SARS-CoV-2 variants were 5,512 ± 3, 5,447 ± 3, 6,091 ± 3, 6,333 ± 3, 6,129 ± 3, 6,733 ± 3, 5,964 ± 3, 6,709 ± 3, and 6,349 ± 3, respectively, and the extended bases were A, T, T, A, G, A, G, C, and G (Fig. 1). All mutation types were identified correctly using mPCR-MS minisequencing methods.

After optimization, the final concentrations of 7 MPEs were 7.0 μM (HV69-70del), 8.09 μM (K417N), 9.09 μM (E484K/Q), 6.87 μM (N501Y), 8.12 μM (D614G), 8.48 μM (P681H/R), and 7.91 μM (L452R).

### Detection limit and specificity of mPCR-MS minisequencing.

**FIG 2:**
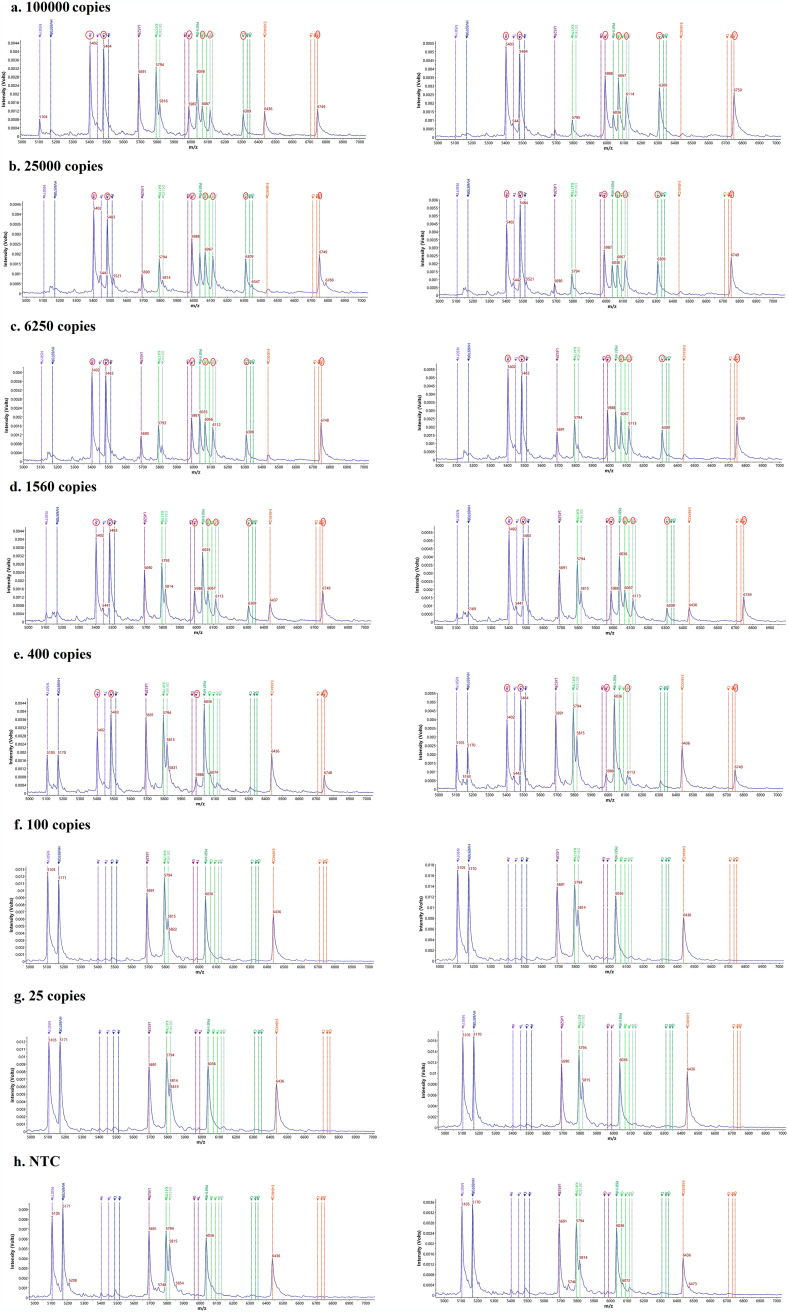
Detection limit of the 9 types of mutations at 7 mutated sites by non-SARS-CoV-2 variants. The left and right sides show two repetitions. NTC, negative control without DNA template.

The extended signals of mass probe pairs 5,483 ± 3/5,512 ± 3 (HV69-70del), 5,402 ± 3/5,447 ± 3 (N501Y), 6,067 ± 3/6,091 ± 3 (K417N), 6,309 ± 3/6,333 ± 3 (P681H), 6,309 ± 3/6,349 ± 3 (P681R), 6,113 ± 3/6,129 ± 3 (D614G), 6,749 ± 3/67,33 ± 3 (E484K), 6,749 ± 3/6,709 ± 3 (E484Q), and 5,988 ± 3/5,964 ± 3 (L452R) were not detected in 21 other respiratory pathogen and non-COVID-19 patient nucleic acids, which showed that the specificity of this method was 100%. The detection limits for HV69-70del, K417N, E484K, N501Y, D614G, P681H, L452R, E484Q, and P681R were 400, 1,560, 400, 400, 400, 1,560, 400, 400, and 1,560 copies, respectively, and the total detection limit for all sites of the mPCR-MS minisequencing was 1,560 copies when using the diluted concentration of the nucleic acids of the SARS-CoV-2 nonvariants (Fig. 2). The mutation sites were detected by synthetic DNA plasmids, and the detection limits of all the sites were between 100 and 400 copies.

### Detection results of clinical nucleic acid specimens.

The threshold cycle (*C_T_*) values of RT real-time PCR (FAM fluorescence signal *C_T_* value of N gene) of nucleic acid samples of 20 COVID-19 patients were 16.04 to 34.95, among which 13 samples with CT within 27 were all positive in the 7 target sites, and three mutations (delta variant) were found. Of the seven specimens with CT values were around 30, some of the sites were detected in 5, and none of the sites were detected in one specimen (Table 1).

**TABLE 1:** Detection results of target sites in COVID-19 patients by mPCR-MS minisequencing^*a*^

No.	Specimen type	*C_T_* value	Detection result								
			N501Y	HV6970R	L452R	K417N	D614G	P681H/R	E484K/Q	mPCR-MS infection type	WGS infection type
1	Sputum	16.04	A	C	T	G	A	C	G	Nonvariant	Nonvariant
2	Sputum	21.05	A	C	T	G	A	C	G	Nonvariant	Nonvariant
3	Sputum	21.05	A	C	T	G	A	C	G	Nonvariant	Nonvariant
4	Sputum	21.12	A	C	T	G	A	C	G	Nonvariant	Nonvariant
5	Sputum	21.60	A	C	T	G	A	C	G	Nonvariant	Nonvariant
6	Sputum	22.19	A	C	T	G	A	C	G	Nonvariant	Nonvariant
7	Sputum	22.24	A	C	T	G	A	C	G	Nonvariant	Nonvariant
8	Sputum	26.31	A	C	T	G	A	C	G	Nonvariant	Nonvariant
9	Sputum	26.47	A	C	T	G	A	C	G	Nonvariant	Nonvariant
10	Sputum	26.89	A	C	T	G	A	C	G	Nonvariant	Nonvariant
11	Sputum	30.35	No call	C	No call	No call	No call	No call	No call	Nonvariant	Nonvariant
12	BALF	30.89	A	C	No call	No call	No call	No call	No call	Nonvariant	Nonvariant
13	Sputum	31.64	A	C	No call	No call	No call	No call	No call	Nonvariant	Nonvariant
14	BALF	32.34	A	C	No call	No call	No call	No call	No call	Nonvariant	Nonvariant
15	Sputum	34.63	A	C	No call	No call	No call	No call	No call	Nonvariant	Nonvariant
16	Sputum	34.95	No call	No call	No call	No call	No call	No call	No call	Negative	Nonvariant
17	NPS	18.56	A	C	**G**	G	**G**	**G**	G	Delta variant	Delta variant
18	NPS	19.71	A	C	**G**	G	**G**	**G**	G	Delta variant	Delta variant
19	NPS	29.91	A	C	No call	No call	No call	No call	No call	Indeterminacy	Delta variant
20	NPS	25.67	A	C	**G**	G	**G**	**G**	G	Delta variant	Delta variant

a Bold indicates mutated base. “Indeterminacy” means that SARS-CoV-2 infection could be determined but the mutation type could not be determined. BALF, bronchoalveolar lavage fluid; NPS, nasopharyngeal swab.

## DISCUSSION

**FIG 3:**
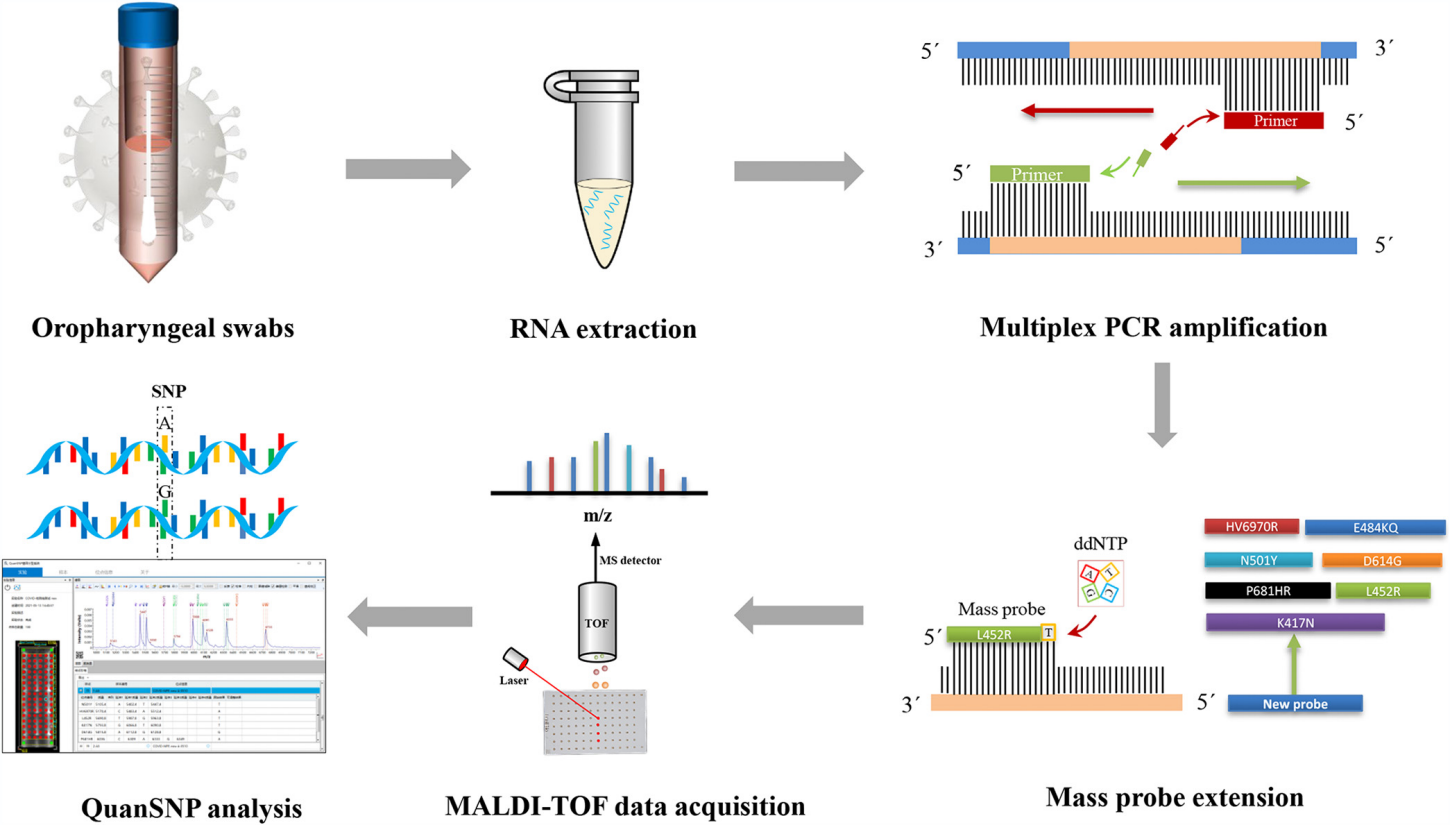
Workflow of mPCR-MS minisequencing for SARS-CoV-2 variant detection.

In view of the emerging SARS-CoV-2 variants, researchers are trying to develop rapid detection methods in addition to genome sequencing. Based on the existing technology, all variants cannot be detected simultaneously by qPCR or CRISPR-Cas13a amplification technology. In this study, we developed a mPCR-MS minisequencing method that can simultaneously detect 9 mutation types in 7 mutated sites in the RBD of spike proteins HV69-70del (Alpha), N501Y (Alpha, Beta, and Gamma), K417N (Beta), P681H (Alpha), D614G (Alpha, Beta, Gamma, Epsilon, Iota, and Delta), E484K (Beta and Gamma), L452R (Delta and Epsilon), P681R (Delta), and E484Q (B.1.617.1 and B1.617.3) to identify SARS-CoV-2 and its mutated variants. The workflow of this method is shown in Fig. 3. Through combination of the results of the nine mutation sites, we can judge whether the detected sample is a SARS-CoV-2 infection, and if it is a SARS-CoV-2 infection, we can also determine which variant strain is present (Table 2).

**TABLE 2:** Interpretation basis of the identification of SARS-CoV-2 variants using mPCR-MS minisequencing

Mutation site^*a*^	Identification results (variants)^*b*^							
	Delta (B.1.617.2)	Alpha (B.1.1.7)	Beta (B.1.351)	Epsilon (B.1.429)	Iota (B.1.526)	Gamma (P.1)	Nonvariants	Non-SARS-CoV-2 infection
HV69-70R del	5,483	5,512*	5,483	5,483	5,483	5,483	5,483	5,170
L452R	5,964*	5,988	5,988	5,964*	5,988	5,988	5,988	5,691
N501Y	5,402	5,447	5,447	5,402	5,447**	5,447*	5,402	5,105
D614G	6,129	6,129	6,129	6,129*	6,129*	6,129*	6,113	5,816
P681H	6,309	6,333*	6,309	6,309	6,309	6,309	6,309	6,036
P681R	6,349*	6,309	6,309	6,309**	6,309	6,309	6,309	6,036
E484K	6,749	6,749	6,733	6,749	6,733*	6,733*	6,749	6,436
E484Q	6,709**	6,749	6,749	6,749**	6,749	6,749	6,749	6,436
K417N	6,067	6,067	6,091*	6,067	6,067	6,091**	6,067	5,794

a Mutation sites detected in this study.

b Single and double asterisks indicate that the mutation sites must be identified and unidentified in the same test, respectively. The mass error of the spectrum peak value in this table is allowed to be less than 500 ppm (±3 Da). The values in the table are the m/z of MPE peaks.

The Indian variant strain B.1.617 lineage includes three main subtypes, B1.617.1, B1.617.2, and B1.617.3. Delta variant (B1.617.2) has been detected in many countries and was believed to spread faster than other variants, and it notably escapes neutralizing monoclonal antibodies and polyclonal antibodies elicited by previous infection with SARS-CoV-2 or by vaccination (22, 23). The method constructed in this study can simultaneously detect 9 mutation sites, which can accurately judge whether the sample is infected by the Delta variant. In the 20 clinical samples, 3 Delta variants were correctly identified according to our judgment rules (Table 2). The nucleic acid load of the samples with real-time PCR *C_T_* values greater than 27 was roughly estimated to be less than 1,000 copies, which was lower than the detection limit of some sites. Therefore, only a portion of sites were detected in six samples with *C_T_* values greater than 27, and one sample was negative. The reliability of the method is verified by the detection of clinical samples, which indicates that the method has clinical application value.

**FIG 4:**
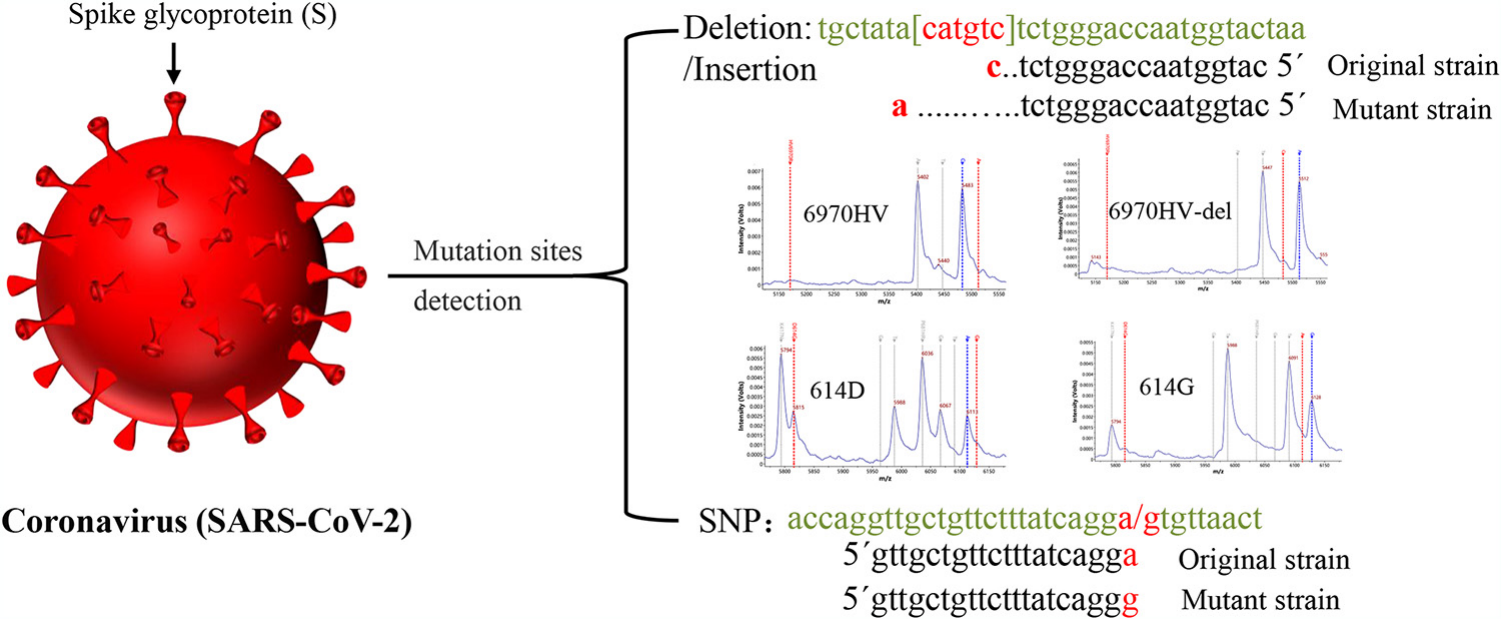
The detection principle of mPCR-MS minisequencing.

This method can not only detect SNP gene mutation but also detect gene variation caused by deletion and insertion according to probe extension. The type of extended base was determined according to the peak value (*m/z*) acquired by MALDI-TOF MS. For example, for the HV69-70del site, if the peak of the detected extension product was 5,483 ± 3, then the extension base was C, indicating that CATGTC (HV69-70R) was not deleted and the detected sample was a non-SARS-CoV-2 variant; if the peak of the detected extension product was 5,512 ± 3, then the extension base was A, indicating that CATGTC was deleted (Fig. 4) and the detected sample was a SARS-CoV-2 variant. If these two characteristic peaks were not detected, the sample was not infected with SARS-CoV-2. The detection of mutation sites through the same principle (Fig. 4).

This method relies on MPE probe detection after PCR amplification and has high specificity. There was no nonspecific identification in various non-SARS-CoV-2 pathogens (9 bacteria and 12 respiratory viruses) or nucleic acid samples from non-COVID-19 patients. The method of detection of mutation sites based on PCR and first-generation sequencing technology has a high requirement for the quantity of PCR amplification products. PCR products with insignificant electrophoretic bands often fail to be sequenced due to insufficient quantity. However, the mPCR-MS minisequencing requires fewer PCR products than sequencing when extending the MPE probe. The PCR products, which are not obvious in electrophoretic bands or even hard to be observed by the naked eye, can also be detected by MALDI-TOF MS after MPE extension. Therefore, for mPCR-MS minisequencing, the detection limit is affected by both multiplex PCR amplification and MPE probe extension, and there are great differences among different mutation sites. For example, D614G and P681H are located in the same PCR amplification fragment. The detection limit of D614G is 400 copies after MPE extension, while that of P681H/R is only 1,560 copies, indicating that the probe extension efficiency of P681H/R is much lower than that of D614G, which is closely related to the base sequence near the detection site of P681H/R. Although the detection limit of the mutant site of synthetic plasmid is lower (100 to 400 copies), considering the lack of RNA virus reverse transcription step in the process of plasmid DNA amplification, we still use the lowest detection limit (LDL) of nucleic acid amplification of SARS-CoV-2-infected samples (1,560 copies) as the LDL of this method.

PCR coupled with first-generation sequencing technology is only suitable for the detection of multiple mutation sites in a nucleic acid sequence of less than 600 bp and cannot be used for the detection of all mutation sites of SARS-CoV-2 (2,000 bp). qPCR is only suitable for the detection of single mutation sites. WGS is time-consuming and high cost and has high technical requirements, so it is only suitable for mutation site analysis in important samples and not suitable for routine sample screening. This method constructed in this study is very suitable for the detection of multiple mutation sites of SARS-CoV-2 variants. In addition, this method is open and extensible and can be used in a high-throughput manner (96 samples can be detected within 7 h), easily allowing the addition of new mutation sites as needed to identify and track new SARS-CoV-2 variants as they emerge.

This study developed a novel strategy for detecting SARS-CoV-2 variants. However, this method has some limitations. First, the 3′-terminal sequence of the extension probe is fixed, and the quality of the probe is greatly influenced by the base sequence near the detection site, which can lead to the inefficient detection in extreme cases. Second, in some HV69-70del mutants, in addition to six bases (CATGTC), the front base of these six bases also had an A-C mutation. The extension probe of HV69-70del could not effectively distinguish the A-C mutation, which does not affect the accuracy for the other six SNP sites. Third, the number of clinical specimens used in this study is limited, and the number of validation specimens needs to be further increased.

As a simple screening assay for monitoring the emergence and spread of these SARS-CoV-2 variants, mPCR-MS minisequencing may be helpful for implementing public health strategies to counter these and future mutation strains.

## MATERIALS AND METHODS

### Specimens and nucleic acids of SARS-CoV-2.

The nucleic acid of the SARS-CoV-2 clinical isolate (nonvariant) was obtained from the Institute of National Institute for Communicable Disease Control and Prevention, Chinese Center for Disease Control and Prevention (ICDC). The S gene mutation plasmids (plasmid 1, containing the HV69-70del, K417N, E484K, N501Y, D614G, and P681H mutations, and plasmid 2, containing the L452R, E484Q, and P681R mutations) of SARS-CoV-2 variants (Alpha, Beta, Iota, Epsilon, Gamma, and Delta) were synthesized by Sangon Biotech (Shanghai, China). The nucleic acids of 21 common respiratory tract pathogens, including Mycoplasma pneumoniae (ATCC 29342), Escherichia coli (ATCC 11229), Streptococcus pneumoniae (ATCC 49619), Staphylococcus aureus (ATCC 29213), Legionella pneumophila (clinical isolate), Mycobacterium tuberculosis (clinical isolate), Pseudomonas aeruginosa (clinical isolate), Haemophilus influenzae (clinical isolate), Neisseria meningitidis (clinical isolate), influenza A virus, influenza B virus, parainfluenza viruses I, II, III, and IV, adenovirus, rhinovirus, coronaviruses 229E, OC43, and NL63, and respiratory syncytial virus, and non-COVID-19 patient sputum and bronchoalveolar lavage fluid specimens were selected for specific validation of this method. The nucleic acids of 20 clinical samples of COVID-19 patients (kept at the ICDC) were used to verify the accuracy of this method. All the specimens collected from COVID-19 patients were collected with complete informed-consent procedures. This study was approved by the ethics committee of the ICDC.

### Design of amplification primers and extension probe for the variant sites.

Three sets of PCR primers were designed to amplify the related fragments of the nine mutation types (Table 3). The S-F1/R1 amplification product contained one mutation type (HV69-70del), the S-F2/R2 amplification product contained five mutation types (K417N, E484K, E484Q, N501Y, and L452R), and the S-F3/R3 amplification product contained three mutation types (D614G, P681H, and P681R). The lengths of primers ranged from 19 bp to 22 bp, and the molecular weight of each primer was made more than 9 kDa (beyond the mass range of the mass extension probe) by adding a 10-bp fixed sequence (ACGTTGGATG) to the 5′ end of each primer. MALDI-TOF MS quality difference probes for mutation sites were designed by using the IntelliBio genetic locus analysis software (V2.0, IntelliBio, China) (Table 3). The molecular weight of the mass probe was required to be 4 to 9 kDa and the length was 17 to 28 bp, and a minimal difference between the probe molecular weights was set for 20 Da.

**TABLE 3:** Primer sequences for SARS-CoV-2 variant target site amplification and MPE probes for target site detection

Target site	Multiple PCR^*a*^		Mass probe extension					
	Forward primer sequence	Reverse primer sequence	Mass probe sequence	Mass probe mass (Da)	Extension base of nonvariant	Extended mass of MPE of nonvariant (Da)	Extension base of variant	Extended mass of MPE of variant (Da)
N501Y	acgttggatgTGCTTTACTAATGTCTATGC	acgttggatgTAGGTCCACAAACAGTTGC	ATGGTTTCCAACCCACT	5,105.4	A	5,402.4	T	5,447.4
HV6970R	acgttggatgCTGCATACACTAATTCTTTC	acgttggatgAATAAGTAGGGACTGGGT	GTACCATTGGTCCCAGA	5,170.4	C	5,483.4	A	5,512.4
L452R	acgttggatgTGCTTTACTAATGTCTATGC	acgttggatgTAGGTCCACAAACAGTTGC	CTTCCTAAACAATCTATAC	5,690.8	T	5,987.8	G	5,963.8
K417N	acgttggatgTGCTTTACTAATGTCTATGC	acgttggatgTAGGTCCACAAACAGTTGC	TATAATTATAATCAGCAAT	5,793.8	G	6,066.8	T	6,090.8
D614G	acgttggatgCTGATGCTGTCCGTGATC	acgttggatgGGGTATGGCAATAGAGTTA	GTTGCTGTTCTTTATCAGG	5,815.8	A	6,112.8	G	6,128.8
P681H	acgttggatgCTGATGCTGTCCGTGATC	acgttggatgGGGTATGGCAATAGAGTTA	TCAGACTCAGACTAATTCTC	6,036.0	C	6,309.0	A	6,333
E484K	acgttggatgTGCTTTACTAATGTCTATGC	acgttggatgTAGGTCCACAAACAGTTGC	AGCACACCTTGTAATGGTGTT	6,436.2	G	6,749.2	A	6,733.2
P681R	acgttggatgCTGATGCTGTCCGTGATC	acgttggatgGGGTATGGCAATAGAGTTA	TCAGACTCAGACTAATTCTC	6,036.0	C	6,309.0	G	6,349.0
E484Q	acgttggatgTGCTTTACTAATGTCTATGC	acgttggatgTAGGTCCACAAACAGTTGC	AGCACACCTTGTAATGGTGTT	6,436.2	G	6,749.2	C	6,709.2

a The 10-bp fixed sequence added to the 5′ end of the primer is indicated by lowercase.

### Establishment of mPCR-MS minisequencing method.

SARS-CoV-2 nonvariants and S gene mutant plasmids of SARS-CoV-2 variants were used as templates to construct the analysis method. Nucleic acid-free water served as the blank control. The specific steps were as follows. (i) For multiplex PCR amplification, the mutated gene fragment was amplified by an AgPath-ID one-step RT-PCR kit. (ii) For shrimp alkaline phosphatase (SAP) digestion, PCR products were treated with SAP to eliminate the free deoxynucleoside triphosphates (dNTPs). (iii) For mass probe extension (MPE), the purified PCR products were added with mixed MPE probes for single base extension, and the mutation sites were detected. (iv) For desalination and purification, an appropriate amount of resin was added to the extension product, turned over and mixed for 30 min, and centrifuged, and the supernatant was subjected to MALDI-TOF MS.

### SNP identification and data analysis by MALDI-TOF MS.

3-Hydroxypyridine-2-carboxylic acid (3-HPA; 0.9 μl) was dropped at the center of the sample target. After drying, the matrix was covered with 0.3 μl of purified supernatant. Then the samples were subjected to testing after crystallization. The data were acquired from the QuanTOF I system (Intelligene Biosystems, Qingdao, China). The parameters and data analysis were previously described (21).

### Determination of the detection limit and specificity.

The lowest detection limit (LDL) of PCR-MALDI-TOF MS was determined by using RNA of SARS-CoV-2 nonvariants and the mutant DNA plasmids of the S gene. The original concentration of nucleic acids was quantified (Qubit 3.0) and diluted in 7 concentration gradients. The specificity of this method was validated by 21 kinds of nucleic acids of the other respiratory pathogens (9 bacteria and 12 respiratory viruses) and human sputum and bronchoalveolar lavage fluid nucleic acid extraction.

### Nucleic acid validation of COVID-19 patient specimens using mPCR-MS minisequencing.

The nucleic acid of specimens of 20 COVID-19 patients was used to test the accuracy of this method. These specimens were also detected with a 2019-nCoV RT real-time PCR detection kit (Da An Gene Co., Ltd.) and WGS.

## References

[1] Tang JW, Tambyah PA, Hui DS. 2021. Emergence of a new SARS-CoV-2 variant in the UK. J Infect 82:e27–e28. doi:10.1016/j.jinf.2020.12.024. PMC7834693 33383088

[2] Cascella M, Rajnik M, Aleem A, Dulebohn SC, Di Napoli R. 2021. Features, evaluation, and treatment of coronavirus (COVID-19). StatPearls, Treasure Island, FL. 32150360

[3] Galloway SE, Paul P, MacCannell DR, Johansson MA, Brooks JT, MacNeil A, Slayton RB, Tong S, Silk BJ, Armstrong GL, Biggerstaff M, Dugan VG. 2021. Emergence of SARS-CoV-2 B.1.1.7 lineage—United States, December 29, 2020–January 12, 2021. MMWR Morb Mortal Wkly Rep 70:95–99. doi:10.15585/mmwr.mm7003e2. 33476315 PMC7821772

[4] Zhou D, Dejnirattisai W, Supasa P, Liu C, Mentzer AJ, Ginn HM, Zhao Y, Duyvesteyn HME, Tuekprakhon A, Nutalai R, Wang B, Paesen GC, Lopez-Camacho C, Slon-Campos J, Hallis B, Coombes N, Bewley K, Charlton S, Walter TS, Skelly D, Lumley SF, Dold C, Levin R, Dong T, Pollard AJ, Knight JC, Crook D, Lambe T, Clutterbuck E, Bibi S, Flaxman A, Bittaye M, Belij-Rammerstorfer S, Gilbert S, James W, Carroll MW, Klenerman P, Barnes E, Dunachie SJ, Fry EE, Mongkolsapaya J, Ren J, Stuart DI, Screaton GR. 2021. Evidence of escape of SARS-CoV-2 variant B.1.351 from natural and vaccine-induced sera. Cell 184:2348–2361.e6. doi:10.1016/j.cell.2021.02.037. 33730597 PMC7901269

[5] Khan A, Zia T, Suleman M, Khan T, Ali SS, Abbasi AA, Mohammad A, Wei DQ. 2021. Higher infectivity of the SARS-CoV-2 new variants is associated with K417N/T, E484K, and N501Y mutants: an insight from structural data. J Cell Physiol 236:7045–7057. doi:10.1002/jcp.30367. 33755190 PMC8251074

[6] Hou YJ, Chiba S, Halfmann P, Ehre C, Kuroda M, Dinnon KH, III, Leist SR, Schafer A, Nakajima N, Takahashi K, Lee RE, Mascenik TM, Graham R, Edwards CE, Tse LV, Okuda K, Markmann AJ, Bartelt L, de Silva A, Margolis DM, Boucher RC, Randell SH, Suzuki T, Gralinski LE, Kawaoka Y, Baric RS. 2020. SARS-CoV-2 D614G variant exhibits efficient replication ex vivo and transmission in vivo. Science 370:1464–1468. doi:10.1126/science.abe8499. 33184236 PMC7775736

[7] Volz E, Hill V, McCrone JT, Price A, Jorgensen D, O’Toole A, Southgate J, Johnson R, Jackson B, Nascimento FF, Rey SM, Nicholls SM, Colquhoun RM, da Silva Filipe A, Shepherd J, Pascall DJ, Shah R, Jesudason N, Li K, Jarrett R, Pacchiarini N, Bull M, Geidelberg L, Siveroni I, COG-UK Consortium, Goodfellow I, Loman NJ, Pybus OG, Robertson DL, Thomson EC, Rambaut A, Connor TR. 2021. Evaluating the effects of SARS-CoV-2 spike mutation D614G on transmissibility and pathogenicity. Cell 184:64–75.e11. doi:10.1016/j.cell.2020.11.020. 33275900 PMC7674007

[8] Laha S, Chakraborty J, Das S, Manna SK, Biswas S, Chatterjee R. 2020. Characterizations of SARS-CoV-2 mutational profile, spike protein stability and viral transmission. Infect Genet Evol 85:104445. doi:10.1016/j.meegid.2020.104445. 32615316 PMC7324922

[9] Bull RA, Adikari TN, Ferguson JM, Hammond JM, Stevanovski I, Beukers AG, Naing Z, Yeang M, Verich A, Gamaarachchi H, Kim KW, Luciani F, Stelzer-Braid S, Eden JS, Rawlinson WD, van Hal SJ, Deveson IW. 2020. Analytical validity of nanopore sequencing for rapid SARS-CoV-2 genome analysis. Nat Commun 11:6272. doi:10.1038/s41467-020-20075-6. 33298935 PMC7726558

[10] Bal A, Destras G, Gaymard A, Stefic K, Marlet J, Eymieux S, Regue H, Semanas Q, d’Aubarede C, Billaud G, Laurent F, Gonzalez C, Mekki Y, Valette M, Bouscambert M, Gaudy-Graffin C, Lina B, Morfin F, Josset L, COVID-Diagnosis HCL Study Group. 2021. Two-step strategy for the identification of SARS-CoV-2 variant of concern 202012/01 and other variants with spike deletion H69-V70, France, August to December 2020. Euro Surveill 26:2100008. doi:10.2807/1560-7917.ES.2021.26.3.2100008. PMC7848679 33478625

[11] Umair M, Ikram A, Salman M, Khurshid A, Alam M, Badar N, Suleman R, Tahir F, Sharif S, Montgomery J, Whitmer S, Klena J. 2021. Whole-genome sequencing of SARS-CoV-2 reveals the detection of G614 variant in Pakistan. PLoS One 16:e0248371. doi:10.1371/journal.pone.0248371. 33755704 PMC7987156

[12] Durner J, Burggraf S, Czibere L, Tehrani A, Watts DC, Becker M. 2021. Fast and cost-effective screening for SARS-CoV-2 variants in a routine diagnostic setting. Dent Mater 37:e95–e97. doi:10.1016/j.dental.2021.01.015. 33551188 PMC7846220

[13] Banada P, Green R, Banik S, Chopoorian A, Streck D, Jones R, Chakravorty S, Alland D. 2021. A simple RT-PCR melting temperature assay to rapidly screen for widely circulating SARS-CoV-2 variants. medRxiv. doi:10.1101/2021.03.05.21252709. PMC8451443 34288729

[14] Wang Y, Zhang Y, Chen J, Wang M, Zhang T, Luo W, Li Y, Wu Y, Zeng B, Zhang K, Deng R, Li W. 2021. Detection of SARS-CoV-2 and its mutated variants via CRISPR-Cas13-based transcription amplification. Anal Chem 93:3393–3402. doi:10.1021/acs.analchem.0c04303. 33511840

[15] Jang KS, Kim YH. 2018. Rapid and robust MALDI-TOF MS techniques for microbial identification: a brief overview of their diverse applications. J Microbiol 56:209–216. doi:10.1007/s12275-018-7457-0. 29492868

[16] Kostrzewa M. 2018. Application of the MALDI Biotyper to clinical microbiology: progress and potential. Expert Rev Proteomics 15:193–202. doi:10.1080/14789450.2018.1438193. 29411645

[17] Rahi P, Prakash O, Shouche YS. 2016. Matrix-assisted laser desorption/ionization time-of-flight mass-spectrometry (MALDI-TOF MS) based microbial identifications: challenges and scopes for microbial ecologists. Front Microbiol 7:1359. doi:10.3389/fmicb.2016.01359. 27625644 PMC5003876

[18] Peng J, Gao L, Guo J, Wang T, Wang L, Yao Q, Zhu H, Jin Q. 2013. Type-specific detection of 30 oncogenic human papillomaviruses by genotyping both E6 and L1 genes. J Clin Microbiol 51:402–408. doi:10.1128/JCM.01170-12. 23152557 PMC3553860

[19] Peng J, Yang F, Xiong Z, Guo J, Du J, Hu Y, Jin Q. 2013. Sensitive and rapid detection of viruses associated with hand foot and mouth disease using multiplexed MALDI-TOF analysis. J Clin Virol 56:170–174. doi:10.1016/j.jcv.2012.10.020. 23194776

[20] Sjoholm MI, Dillner J, Carlson J. 2008. Multiplex detection of human herpesviruses from archival specimens by using matrix-assisted laser desorption ionization-time of flight mass spectrometry. J Clin Microbiol 46:540–545. doi:10.1128/JCM.01565-07. 18094141 PMC2238100

[21] Zhao F, Zhang J, Wang X, Liu L, Gong J, Zhai Z, He L, Meng F, Xiao D. 2021. A multisite SNP genotyping and macrolide susceptibility gene method for Mycoplasma pneumoniae based on MALDI-TOF MS. iScience 24:102447. doi:10.1016/j.isci.2021.102447. 33997713 PMC8105657

[22] Planas D, Veyer D, Baidaliuk A, Staropoli I, Guivel-Benhassine F, Rajah MM, Planchais C, Porrot F, Robillard N, Puech J, Prot M, Gallais F, Gantner P, Velay A, Le Guen J, Kassis-Chikhani N, Edriss D, Belec L, Seve A, Courtellemont L, Pere H, Hocqueloux L, Fafi-Kremer S, Prazuck T, Mouquet H, Bruel T, Simon-Loriere E, Rey FA, Schwartz O. 2021. Reduced sensitivity of SARS-CoV-2 variant Delta to antibody neutralization. Nature 596:276–280. doi:10.1038/s41586-021-03777-9. 34237773

[23] Lopez Bernal J, Andrews N, Gower C, Gallagher E, Simmons R, Thelwall S, Stowe J, Tessier E, Groves N, Dabrera G, Myers R, Campbell CNJ, Amirthalingam G, Edmunds M, Zambon M, Brown KE, Hopkins S, Chand M, Ramsay M. 2021. Effectiveness of Covid-19 vaccines against the B.1.617.2 (Delta) variant. N Engl J Med 385:585–594. doi:10.1056/NEJMoa2108891. 34289274 PMC8314739

